# Bee Bread Protects Small Intestinal Mucosa and Modulates Local Redox Responses in Streptozotocin‐Induced Diabetic Rats

**DOI:** 10.1002/mnfr.70565

**Published:** 2026-08-18

**Authors:** Ebru Karadag Sari, Buket Bakιr, Sükran Yediel Aras, Sevda Elis Yιldιz, Arzu Gezer

**Affiliations:** ^1^ Faculty of Veterinary Medicine Department of Histology and Embryology Kafkas University Kars Türkiye; ^2^ Faculty of Veterinary Medicine Department of Histology and Embryology Tekirdağ Namık Kemal University Tekirdağ Türkiye; ^3^ Faculty of Health Sciences Department of Midwifery Kafkas University Kars Türkiye; ^4^ Vocational School of Health Services Atatürk University Erzurum Türkiye; ^5^ Graduate School of Natural and Applied Sciences Pharmaceutical Research and Development Atatürk University Erzurum Türkiye

**Keywords:** apitherapy, Bee Bread, diabetes, intestine, Mn‐SOD

## Abstract

Bee Bread (BB), a fermented bee product rich in antioxidant compounds, exerts protective effects against diabetes‐associated small intestinal damage. This study investigated the effects of BB on small intestinal morphology, mucosal integrity, and antioxidant enzyme activity in streptozotocin (STZ)‐induced diabetic rats. Forty *Wistar albino* rats were randomly divided into five groups: control, sham (50 mg/kg sodium citrate), diabetes (DM; 50 mg/kg STZ), BB (100 mg/kg), and Diabetes + Bee Bread (DM+BB). After 15 days, intestinal tissues were examined using histopathological, immunohistochemical (IHC), and scanning electron microscopy (SEM) analyses. Diabetic rats exhibited hyperglycemia (363.63 ± 14.64 mg/dL vs. 97.25 ± 2.71 mg/dL in controls at day 15; *p* < 0.001), weight loss, villus elongation, epithelial desquamation, and mucosal disorganization. BB supplementation reduced blood glucose levels (approximately 15%–16% in the DM+BB group), attenuated weight loss, and preserved small intestinal architecture, with significant differences in villus length among groups (*p* < 0.05). Mn‐SOD immunoreactivity showed no significant differences among groups (*p* > 0.05), although redistribution was observed in diabetic tissues. CAT immunoreactivity remained weak and unchanged across groups. SEM analysis confirmed improved villus architecture and preserved goblet cell morphology following BB treatment. BB attenuated diabetes‐induced small intestinal injury and preserved intestinal mucosal architecture, findings that may be associated with localized adaptive redox responses.

## Graphical abstract

In streptozotocin‐induced diabetic rats, bee bread supplementation attenuated diabetes‐associated intestinal mucosal alterations and modulated local redox responses. Treatment reduced blood glucose levels and influenced the immunoreactivity patterns of Mn‐SOD and CAT, supporting a protective role of bee bread in maintaining small intestinal mucosal integrity and redox homeostasis under diabetic conditions.

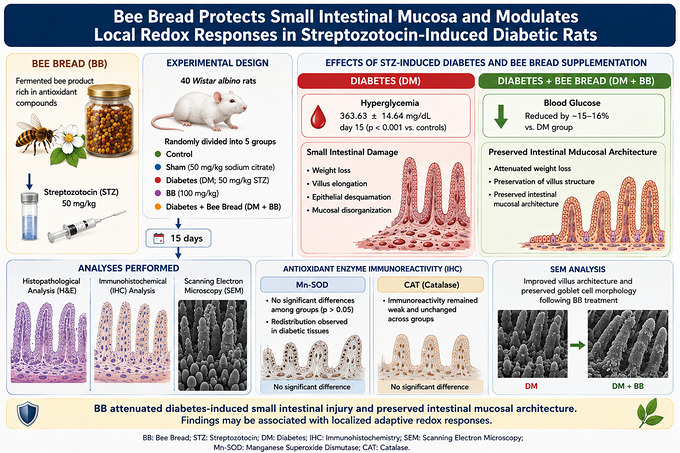

## 1 Introduction

Bee products, including honey, Bee Bread (BB), propolis, royal jelly, and pollen, have long been recognized for their bioactive properties and health benefits [1]. Among these, BB is a natural fermented product formed through the microbial transformation of pollen mixed with honey and bee secretions. This fermentation process increases nutrient bioavailability and enriches the product with bioactive compounds including phenolic acids, flavonoids, amino acids, vitamins, and antioxidant‐related enzymes [2, 3]. Compared with raw pollen, BB exhibits higher antioxidant capacity and improved digestibility, supporting its potential as a functional food in oxidative stress‐related disorders [2].

Diabetes mellitus (DM) is a chronic metabolic disease characterized by persistent hyperglycemia and progressive multi‐organ complications. Beyond metabolic dysregulation, DM is strongly associated with diabetic enteropathy, which involves structural and functional deterioration of the small intestinal mucosa, including epithelial damage, goblet cell dysfunction, impaired barrier integrity, and disrupted villus architecture [4]. In addition to these degenerative changes, intestinal adaptation responses such as villus elongation and mucosal remodeling may also occur under chronic hyperglycemic stress, reflecting a dysregulated repair process rather than true tissue recovery. These alterations are primarily driven by excessive reactive oxygen species (ROS) production, mitochondrial dysfunction, and disruption of intestinal redox homeostasis. Within this context, manganese superoxide dismutase (Mn‐SOD), the key mitochondrial antioxidant enzyme, plays a central role in regulating oxidative stress and maintaining mitochondrial integrity. Changes in its cellular distribution, particularly within crypt epithelial compartments, may therefore reflect localized mitochondrial oxidative stress rather than adaptive protection [5, 6].

BB has been reported to exert strong antioxidant and cytoprotective effects due to its rich content of phenolic compounds, flavonoids, and enzymatic antioxidants. These compounds are known to modulate oxidative stress responses, enhance endogenous antioxidant systems, and support epithelial barrier integrity [7, 8]. In addition, the fermented structure of BB may increase intestinal bioaccessibility and enable direct interaction with mucosal tissues [9]. Despite these properties, its effects on intestinal structural integrity and compartment‐specific antioxidant responses under diabetic conditions remain insufficiently characterized.

Importantly, previous studies have largely focused on systemic metabolic or general antioxidant effects of BB, while its impact on intestinal ultrastructure, segment‐specific mucosal remodeling, and Mn‐SOD/CAT localization in diabetic enteropathy remains poorly understood. In particular, there is a lack of integrated histological, ultrastructural, and immunohistochemical evidence linking BB to intestinal redox regulation in diabetes.

Therefore, the present study aimed to investigate the effects of BB supplementation on intestinal morphology, mucosal integrity, ultrastructural organization, and antioxidant enzyme responses in streptozotocin‐induced diabetic rats. Histopathological analysis, immunohistochemistry (Mn‐SOD and CAT), and scanning electron microscopy were employed to evaluate structural and redox‐related alterations across intestinal segments.

We hypothesized that BB would attenuate diabetes‐induced intestinal injury by modulating mitochondrial redox homeostasis and preserving mucosal barrier integrity, rather than by directly normalizing blood glucose levels. Importantly, we further propose that the protective effects of BB may be associated with localized modulation of Mn‐SOD‐related mitochondrial redox responses within intestinal epithelial compartments.

## 2 Materials and Methods

### 2.1 Preparation of BB Extract

Dried powdered BB obtained from *Apis mellifera* hives (Tekirdağ, Türkiye) was used for extract preparation [2]. The BB (10 g) was mixed with 100 mL of a water–ethyl acetate solution (70:30, *v/v*) and homogenized in an orbital mixer at 5000 rpm for 30 min. This solvent system was selected because it provides intermediate polarity, allowing efficient extraction of both hydrophilic phenolic acids and moderately lipophilic flavonoids while minimizing the extraction of non‐phenolic matrix components. The mixture was then centrifuged at 3500 rpm for 45 min at 4°C, and the resulting supernatant was filtered through a 0.45 µm PTFE membrane, according to a modified method described by Birinci et al. [10]. The modifications included using a water–ethyl acetate solvent and adjusting the centrifugation conditions to optimize phenolic extraction. The filtered extract was aliquoted and stored at −20°C, protected from light, until further use. The extraction yield was calculated as 18% (*w/w*). For chemical characterization, the total phenolic content was determined using the Folin–Ciocalteu method and expressed as mg of gallic acid equivalents (GAE)/g of extract [11]. The total flavonoid content was measured using the aluminum chloride colorimetric method and expressed as mg of quercetin equivalents (QE)/g of extract [12]. Additionally, HPLC analysis was performed to identify key phenolic and flavonoid compounds [13]. The stock solution of the extract was formulated at a concentration of 10 mg/mL, and animals received 100 mg/kg body weight via oral gavage [7].

### 2.2 Animals and Experimental Model

Male *Wistar albino* rats (*n* = 40, 8 per group), aged 4 months, were obtained from the university's experimental animal facility. Following a 1‐day acclimatization period, during which animals were allowed to adapt to laboratory conditions without any intervention, the animals were randomly assigned to five experimental groups (*n* = 8 per group) using a computer‐generated randomization sequence. The rats were housed in standard cages under controlled environmental conditions (25°C ± 2°C, 60%–65% relative humidity, 12 h light/dark cycle) with ad libitum access to standard chow (Table 1) and water throughout the experimental period.

**TABLE 1:** Composition of the standard chow provided to *Wistar albino* rats (g/kg).

Ingredient	g/kg
Cornmeal	600
Soybean meal	200
Wheat bran	100
Soybean oil	30
Vitamin‐mineral mix	20
Choline bitartrate	10
Total	960

*notes*: Values are approximate; the total is close to 1000 g. The chow provided complete nutrition for rats throughout the 15‐day experimental period.

The experimental protocol lasted 15 days.

On day 1, diabetes was induced in the relevant groups by a single intraperitoneal (i.p.) injection of STZ (50 mg/kg), freshly dissolved in 0.1 M citrate buffer (pH 4.5) [14]. Control animals received no intervention, while sham animals received an equivalent volume of vehicle solution (0.1 M citrate buffer, pH 4.5) via the same route [14].

On day 3, fasting blood glucose levels were measured after an 8 h fasting period. Rats with blood glucose levels > 250 mg/dL were considered diabetic and included in the diabetic groups [14].

From day 3 to day 15 (12 consecutive days), oral gavage treatments were administered as follows:

Control Group: No intervention was performed throughout the experimental period.

Sham Group: Rats received a single intraperitoneal injection of vehicle solution (0.1 M citrate buffer, pH 4.5) on day 1. In addition, to control for handling and oral gavage‐related stress, animals received saline (0.5 mL) by oral gavage once daily for 12 consecutive days [15].

DM Group: Diabetes was induced on day 1 by a single intraperitoneal injection of STZ (50 mg/kg). No further treatment was administered [16, 17].

BB Group: Rats received BB extract (100 mg/kg/day) by oral gavage once daily from day 3 to day 15 [7].

DM+BB Group: Diabetes was induced as described above. Following the confirmation of diabetes on day 3, diabetic rats received BB extract (100 mg/kg/day) by oral gavage once daily from day 3 to day 15.

All animals were maintained under identical environmental and handling conditions throughout the study. Fasting blood glucose levels were measured on days 0, 3, 7, and 15 via tail vein sampling after an 8 h fasting period.

At the end of the experimental period (day 15), all animals were fasted overnight and deeply anesthetized by intraperitoneal injection of thiopental sodium (20 mg/kg) combined with inhalation anesthesia using 5% sevoflurane. Adequate anesthesia was confirmed by the absence of the pedal withdrawal reflex. Euthanasia was performed with a high dose of thiopental sodium (50 mg/kg, i.p.). Small intestinal tissues were immediately excised under sterile conditions and fixed in 10% neutral buffered formalin for subsequent histopathological and immunohistochemical analyses.

### 2.3 Histopathological Examination

Small intestinal tissue samples were stabilized in 10% formaldehyde solution for 24 h. Subsequently, the samples were dehydrated in a graded ethanol series, cleared with xylene, and encased in paraffin. Paraffin blocks were sliced at 5 µm thickness using a rotary microtome (LEICA) and stained with Mallory's modified triple stain and hematoxylin and eosin (H&E) to evaluate the general histologic structure of the tissues [18]. Histopathological evaluations were conducted by a pathologist blinded to the experimental groups. All slides were coded to ensure blind assessment and examined under × 10 and × 40 magnifications, depending on the histological features assessed. Preparations were photographed and analyzed using a light microscope (Olympus BX53, Japan). For semi‐quantitative evaluation, a histopathological scoring system was applied based on the severity of villus damage, epithelial integrity, and mucosal architecture (0 = normal structure, 1 = mild damage, 2 = moderate damage, 3 = severe damage).

### 2.4 IHC Examination

The Streptavidin‐Biotin Peroxidase complex (ABC) method was used to determine the localization of Mn‐SOD and CAT immunoreactivity in intestinal tissues. 5 µm thick cross‐sections were mounted on chrome alum gelatin‐coated slides and subjected to deparaffinization and dehydration. To block endogenous peroxidase activity, sections were incubated in 3% H_2_O_2_ in methanol for 20 min. Antigen retrieval was applied by heating the sections in a sodium citrate buffer (pH 6.0) at 600 watts in a microwave oven for 20 min. Following antigen retrieval, sections were incubated with Blocking Solution A (Invitrogen Histostain Plus Bulk Kit) for 10 min and then treated with primary antibodies Mn‐SOD (Santa Cruz, sc‐133254; 1:500 dilution) and CAT (Santa Cruz, sc‐271358; 1:50 dilution) for 1 h at room temperature. The primary antibodies were used at different dilutions according to the manufacturers’ recommendations and preliminary optimization experiments to obtain optimal staining quality and specificity. Following incubation, sections were treated with a biotinylated secondary antibody for 30 min, followed by Streptavidin‐Peroxidase incubation for 30 min. The antibody reaction was visualized using DAB chromogen solution, and slices were examined under a light microscope. The reaction was stopped by washing with PBS once satisfactory immunoreactivity was observed. Sections were counterstained with modified Gill III hematoxylin, dehydrated, and coverslipped with Entellan [17]. IHC images were evaluated under × 40 magnification, and the scale bar was set at 50 µm.

ImageJ software was used for quantification of positively stained cells. Cell counts were performed by randomly selecting five slides from each group and analyzing four fields per slide, totaling 20 fields per group. Negative control sections were processed identically except for omission of the primary antibodies. All slides were coded and evaluated blindly. Immunohistochemical data obtained from ImageJ analysis were considered the primary quantitative endpoint and included in statistical analysis. Semiquantitative scoring was used as a complementary descriptive method to support morphological interpretation and was performed independently by two blinded observers. Immunoreactivity was evaluated semiquantitatively by two independent observers based on staining intensity, scored as follows: no immunoreactivity (−), weak (+), moderate (++), and strong (+++). To reduce subjectivity and improve reproducibility, scoring was performed independently by two blinded observers, and the final score was obtained by consensus. Inter‐observer agreement was assessed to ensure scoring consistency [19].

### 2.5 SEM Examination

Intestine tissue samples were rinsed in 0.1 M PBS and fixed in 3% glutaraldehyde solution for 24 h. Following fixation, samples were dehydrated through a graded acetone series ‘25%, 50%, 70%, and 100%’ and mounted on stubs. The tissue samples were then examined using an FEI Quanta FEG 250 SEM operated in low vacuum mode. The villi, goblet cells, and intestinal mucosa were evaluated. Villus length was measured on 60 individual villi selected by a random sampling method from different regions of each intestinal segment and recorded accordingly [20]. SEM images were evaluated under × 2000 magnification, and the scale bar was set at 50 µm.

### 2.6 Statistical Analysis

Data are presented as mean ± standard error of the mean (SEM). Statistical analyses were performed using GraphPad Prism version 9.0 (GraphPad Software, San Diego, CA, USA). Data normality was assessed using the Shapiro–Wilk test. Differences among groups were analyzed using one‐way analysis of variance (ANOVA), followed by Tukey's post hoc multiple comparison test. *p* < 0.05 was considered statistically significant.

## 3 Results

### 3.1 Body Weight Results

At baseline (day 1), no statistically significant differences were observed among groups (*p* > 0.05). A one‐way ANOVA demonstrated significant differences in body weight among groups over the experimental period (*p* < 0.05). Post‐hoc Tukey analysis revealed significant differences between the Control and Diabetes groups, as well as between the Diabetes and DM+BB groups (*p* < 0.05). BB administration alone did not result in a statistically significant difference in body weight compared with the control or sham groups (*p* > 0.05). However, diabetic animals exhibited a progressive reduction in body weight throughout the experiment, whereas BB supplementation partially attenuated this weight loss (Table 2; *p* < 0.05).

**TABLE 2:** Daily live‐weight comparisons among control, sham, DM, BB, and DM+BB *Wistar albino* rats (mean ± SD).

Day	Control	Sham	DM	BB	DM+BB	*p*
1	343.75 ± 10.26	304.88 ± 4.36	340.75 ± 16.44	315.13 ± 16.50	339.00 ± 9.89	0.114
2	330.50 ± 14.49	308.88 ± 3.45	330.13 ± 15.00	330.13 ± 24.10	315.63 ± 8.16	0.757
3	346.13 ± 14.95	297.88 ± 16.68	332.00 ± 14.29	324.38 ± 24.30	302.38 ± 9.58	0.229
4	349.63 ± 13.78	298.88 ± 17.61	315.00 ± 14.89	314.63 ± 28.05	312.88 ± 6.94	0.356
5	361.00 ± 13.24^a^	316.88 ± 17.81^ab^	298.00 ± 15.33^b^	321.25 ± 26.41^ab^	299.88 ± 8.40^b^	0.094
6	367.63 ± 12.40^a^	325.88 ± 18.79^ab^	286.50 ± 14.50^b^	333.50 ± 26.68^ab^	287.13 ± 8.08^b^	0.010
7	373.38 ± 12.59^a^	334.13 ± 18.65^a^	277.00 ± 15.08^b^	337.00 ± 26.99^a^	281.25 ± 8.31^b^	0.002
8	381.50 ± 13.71^a^	339.25 ± 18.03^a^	258.75 ± 13.33^b^	345.25 ± 25.97^a^	276.50 ± 8.21^b^	0.001
9	385.13 ± 13.70^a^	343.00 ± 18.10^a^	250.63 ± 12.75^b^	352.00 ± 26.03^a^	269.63 ± 8.04^b^	0.001
10	395.38 ± 13.85^a^	345.63 ± 17.96^a^	243.00 ± 12.67^b^	358.13 ± 26.16^a^	272.13 ± 7.97^b^	0.001
11	397.25 ± 14.38^a^	348.50 ± 17.81^a^	240.38 ± 12.63^b^	360.38 ± 26.00^a^	269.38 ± 7.76^b^	0.001
12	400.00 ± 14.05^a^	352.13 ± 17.89^a^	233.25 ± 11.60^b^	362.00 ± 26.04^a^	267.75 ± 7.42^b^	0.001
13	402.38 ± 14.28^a^	354.50 ± 18.06^a^	231.38 ± 11.37^b^	366.50 ± 24.89^a^	264.75 ± 7.03^b^	0.001
14	405.63 ± 14.29^a^	358.38 ± 17.72^a^	229.38 ± 11.29^b^	367.88 ± 24.62^a^	262.25 ± 6.83^b^	0.001
15	411.25 ± 15.93^a^	359.25 ± 17.55^b^	227.50 ± 11.39^c^	369.88 ± 24.37^ab^	257.63 ± 8.27^c^	0.001
*p*	0.001	0.022	0.001	0.842	0.001	—

^a,b,c^Values with different superscript letters within the same row indicate statistically significant differences between groups (*p* < 0.05).

### 3.2 Blood Glucose Levels

There was no statistically significant difference in blood glucose levels between the groups on the 1st day (*p* > 0.05). However, on the 3rd and 7th days, significant differences were observed between the control, sham, and BB groups and the DM and DM+BB groups (*p* < 0.05). By the end of the experiment, no significant differences were found among the control, sham, and BB groups (*p* > 0.05), whereas statistically significant differences were observed both between the DM and DM+BB groups and between these two groups and the control, sham, and BB groups (Table 3).

**TABLE 3:** Blood glucose levels on different days in control, sham, DM, BB, and DM+BB *Wistar albino* rats (mean ± SD).

Day	Control	Sham	DM	BB	DM+BB	*p*
0	96.75 ± 3.21^a^	88.25 ± 2.97^b^	088.13 ± 0.97^b^	92.13 ± 3.18^ab^	094.00 ± 2.22^ab^	0.119
3	96.50 ± 2.68^b^	91.38 ± 2.03^b^	318.88 ± 14.18^a^	94.63 ± 2.09^b^	342.38 ± 17.71^a^	0.001
7	96.88 ± 2.91^b^	95.00 ± 2.32^b^	347.50 ± 13.74^a^	93.00 ± 3.07^b^	321.63 ± 15.54^a^	0.001
15	97.25 ± 2.71^c^	96.00 ± 1.96^c^	363.63 ± 14.64^a^	93.25 ± 2.97^c^	305.75 ± 17.29^b^	0.001
*p*	0.998	0.103	0.001	0.941	0.001	

^a,b,c^Values with different superscript letters within the same row indicate statistically significant differences between groups (*p* < 0.05).

### 3.3 Histopathological Results

**FIGURE 1:**
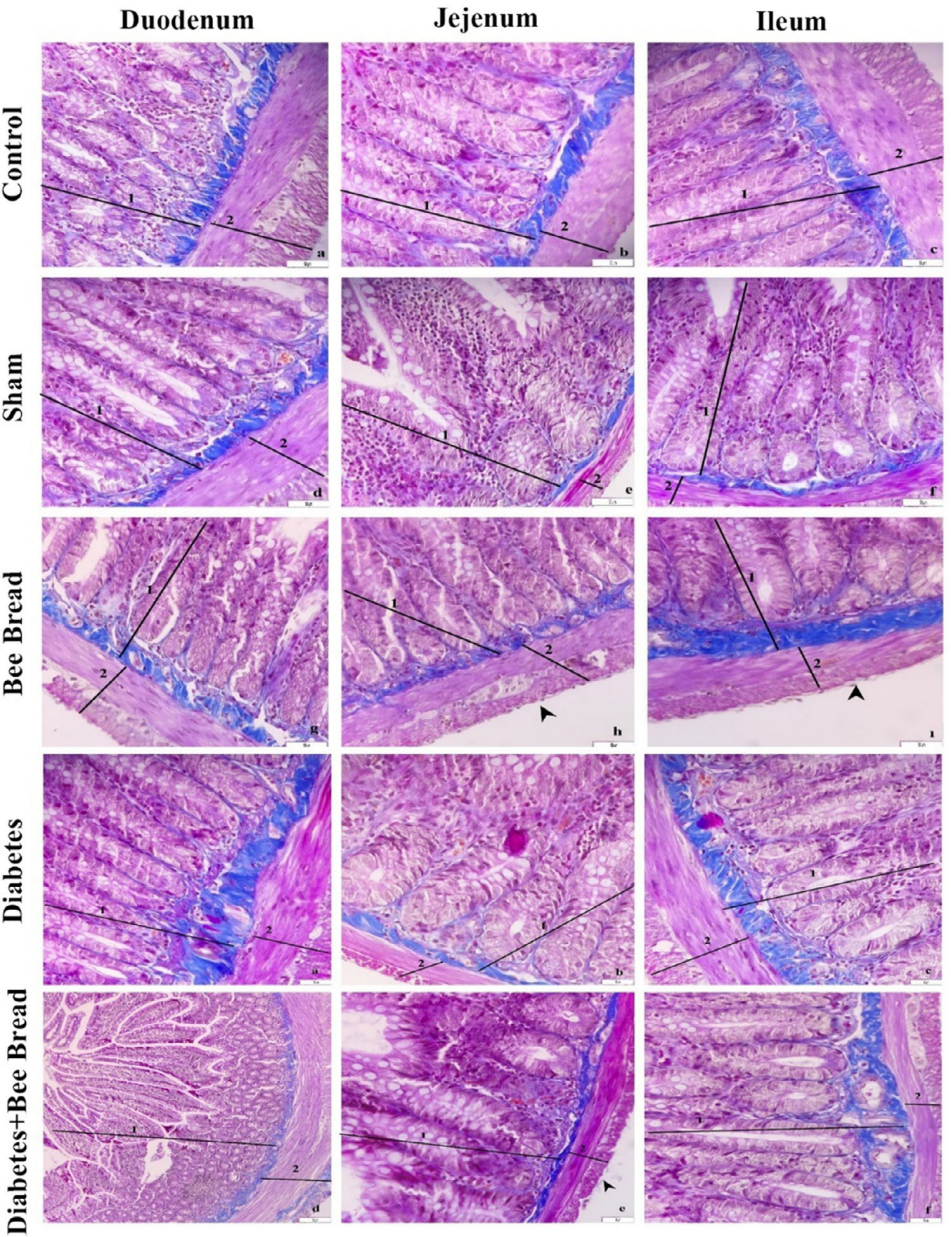
Histological sections of the intestine in control, sham, BB, DM, and DM+BB groups. The small intestinal histological structure appeared normal in the control, sham, and BB groups. In contrast, the DM group exhibited villus degeneration, epithelial desquamation, irregular goblet cell distribution, and disruption of normal mucosal architecture. BB treatment preserved villus architecture and epithelial integrity and reduced the structural disorganization observed in diabetic animals. Mucosa (1), muscularis layer (2), serosa (arrowhead). Triple staining. All images were obtained at × 40 magnification. Scale bar = 50 µm.

**FIGURE 2:**
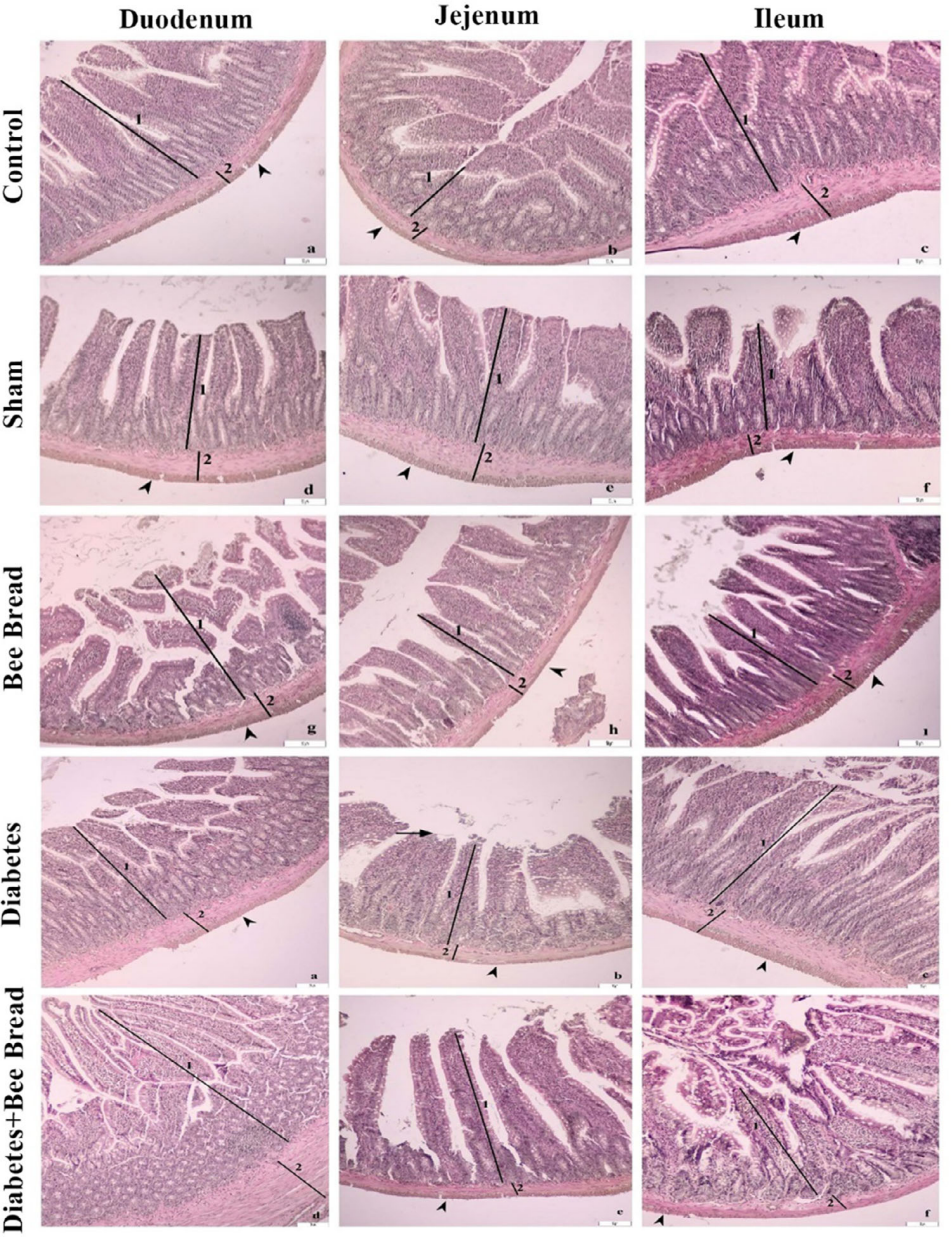
Histological sections of the intestine in control, sham, BB, DM, and DM+BB groups. The small intestinal histological architecture appeared normal in the control, sham, and BB groups. In contrast, the DM group exhibited villus degeneration, epithelial desquamation (arrow), and disruption of normal mucosal organization. BB treatment preserved villus architecture and epithelial integrity and attenuated the histopathological alterations associated with diabetes. Mucosa (1), muscularis layer (2), serosa (arrowhead). H&E staining. All images were obtained at × 10 magnification. Scale bar = 200 µm.

The histological structure of the intestine appeared normal in the control, sham, and BB groups. However, various histopathological and structural alterations were observed in the DM and DM+BB groups. In the jejunal tissue of the DM group, villus degeneration and epithelial desquamation were detected, whereas these changes were not observed in the DM+BB group (Figures 1 and 2). Semi‐quantitative histopathological scoring revealed significantly higher scores in the DM group compared with the control, sham, and BB groups (*p* < 0.05). In contrast, the DM+BB group exhibited significantly lower histopathological scores than the untreated DM group (*p* < 0.05), indicating partial restoration of intestinal morphology.

### 3.4 IHC Results

#### 3.4.1 Mn‐SOD Immunoreactivity

**FIGURE 3:**
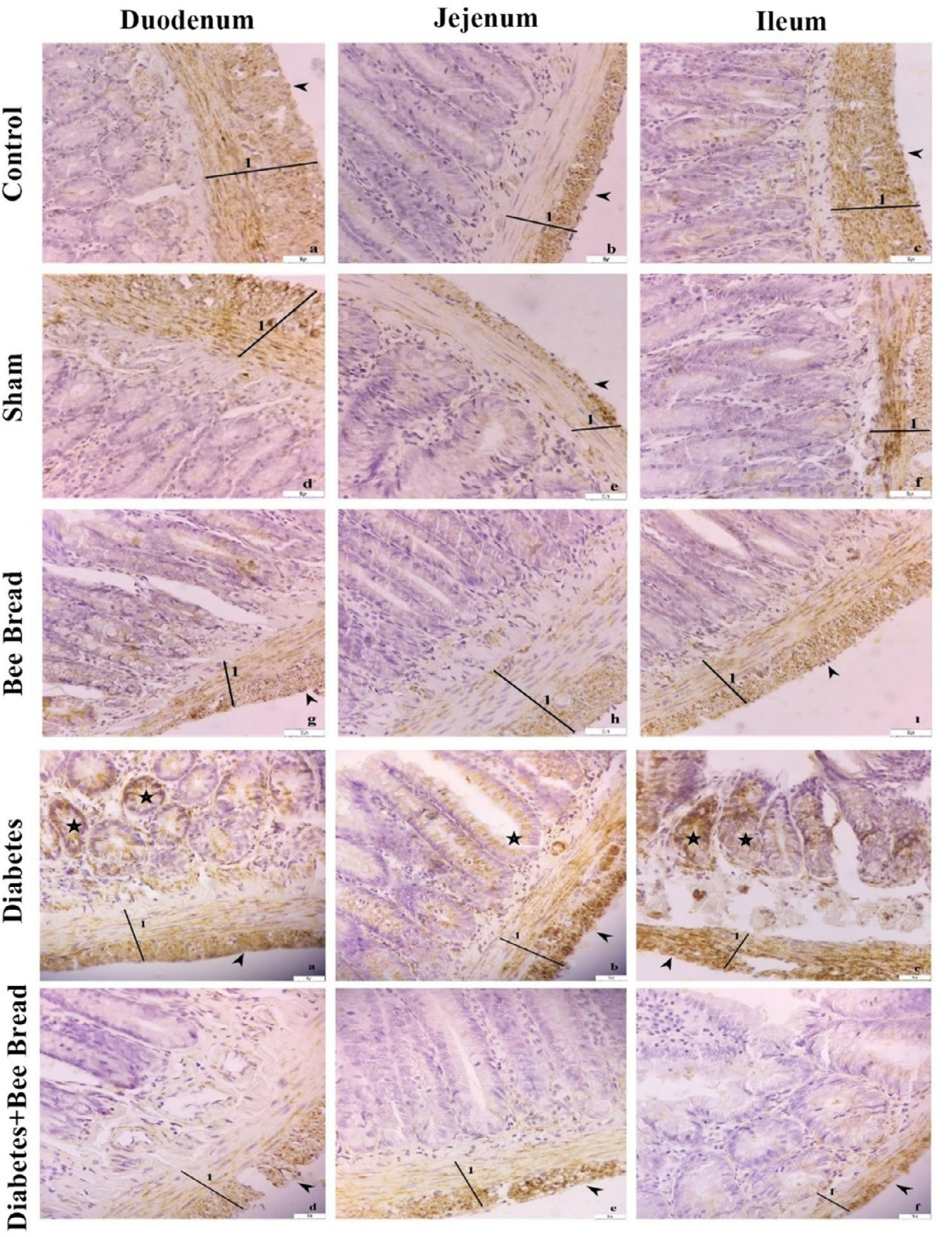
Representative Mn‐SOD immunohistochemical staining in the intestine of control, sham, BB, DM, and DM+BB groups. Mn‐SOD immunoreactivity was observed in the muscularis and serosa layers in the duodenum (a, d, g, a, d), jejunum (b, e, h, b, e), and ileum (c, f, i, c, f). The staining pattern and intensity were comparable among experimental groups, in agreement with the semiquantitative immunoreactivity scores presented in Table 4. Muscularis layer (1), serosa (arrowhead), crypt epithelial cells (asterisk). All images were obtained at × 40 magnification. Scale bar = 50 µm.

Mn‐SOD immunoreactivity was detected in the muscularis and serosa layers of the duodenum, jejunum, and ileum in all experimental groups, showing a similar distribution pattern across groups. In addition to these layers, Mn‐SOD immunoreactivity was also observed in the cytoplasm of crypt epithelial cells in the DM group; however, this localization did not differ in intensity compared with the other groups (Table 4 and Figure 3).

**TABLE 4:** Mn‐SOD immunoreactivity scores in duodenum, jejunum, and ileum tissues of control, sham, DM, BB, and DM+BB *Wistar albino* rats.

Tissue Region	Control	Sham	DM	BB	DM+BB	*F*	*p*
Duodenum	2.93 ± 0.13	2.93 ± 0.13	2.95 ± 0.13	2.93 ± 0.13	2.93 ± 0.13	0.034	0.998
Jejunum	2.95 ± 0.13	2.93 ± 0.15	2.95 ± 0.13	2.93 ± 0.15	2.95 ± 0.13	0.046	0.996
Ileum	2.93 ± 0.15	2.93 ± 0.15	2.93 ± 0.15	2.95 ± 0.13	2.93 ± 0.15	0.065	0.992

Data are expressed as mean ± SD. No statistically significant differences were observed among groups (*p*>0.05).

#### 3.4.2 CAT Immunoreactivity

**FIGURE 4:**
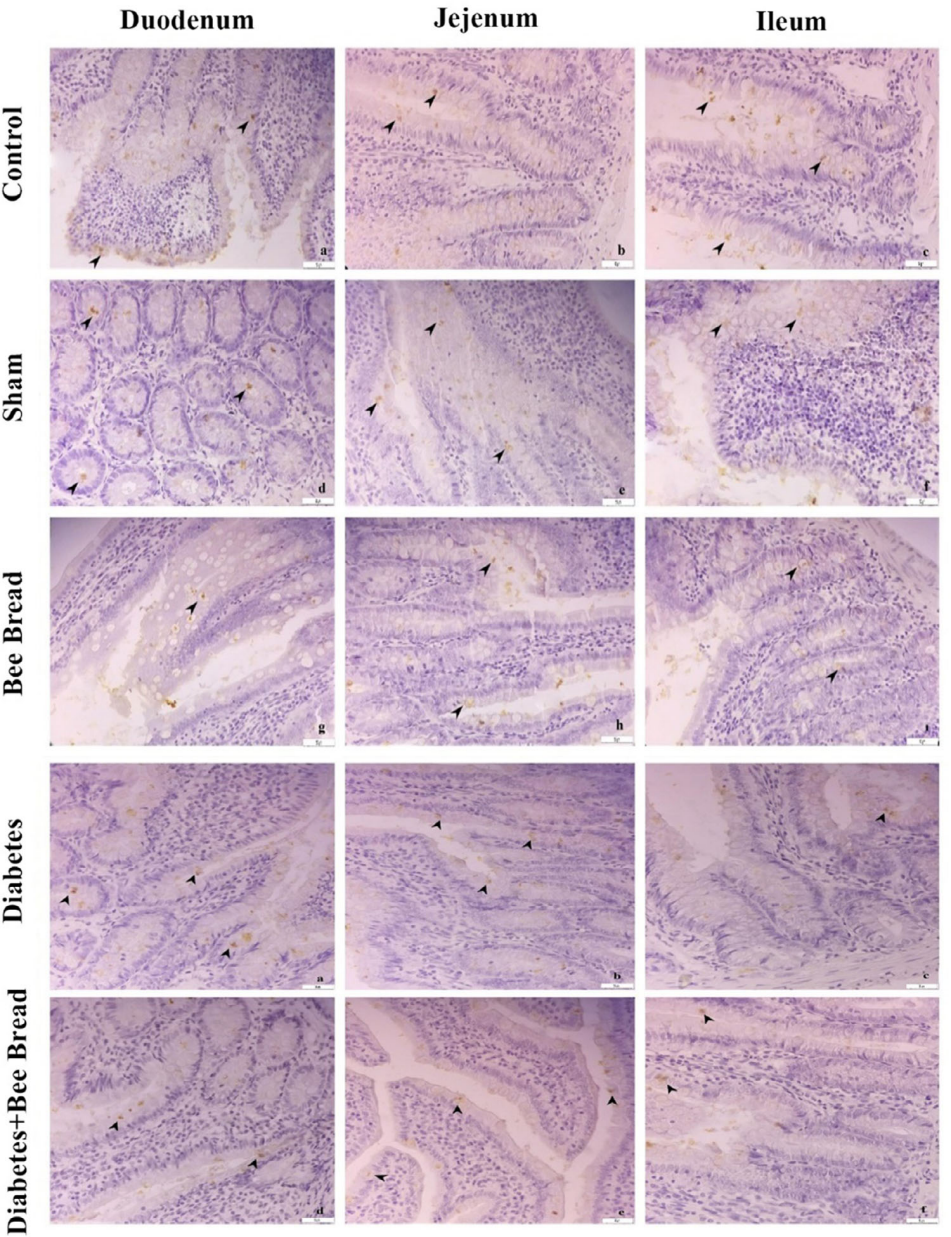
Representative CAT immunohistochemical staining in the intestine of control, sham, BB, DM, and DM+BB groups. CAT immunoreactivity was weak and predominantly localized in the cytoplasm of villus and crypt epithelial cells in the duodenum (a, d, g, a, d), jejunum (b, e, h, b, e), and ileum (c, f, i, c, f) across all groups. No differences in staining intensity or distribution were observed between control, DM, and treatment groups, consistent with the semiquantitative scores presented in Table 5. Villus and crypt epithelial cytoplasm (arrowhead). All images were obtained at × 40 magnification. Scale bar = 50 µm.

Weak CAT immunoreactivity was detected in the cytoplasm of some villi and crypt epithelium in the duodenum, jejunum, and ileum in all groups, with a similar staining pattern among experimental groups (Table 5 and Figure 4).

**TABLE 5:** CAT immunoreactivity scores in duodenum, jejunum, and ileum tissues of control, sham, DM, BB, and DM+BB *Wistar albino* rats.

Tissue Region	Control	Sham	Diabetes	BB	DM+BB	F	*p*
Duodenum	1.00 ± 0.00	1.00 ± 0.00^a^	1.02 ±0.07^a^	1.03 ± 0.09^a^	1.02 ± 0.07^a^	1.39	0.243
Jejunum	1.05 ± 0.10	1.02 ± 0.07^b^	1.00 ±0.00^b^	1.02 ± 0.07^b^	1.05 ± 0.10^b^	1.33	0.264
Ileum	1.01 ± 0.05^c^	1.01 ± 0.05^c^	1.02 ± 0.07^c^	1.02 ± 0.07^c^	1.03 ± 0.09^c^	0.41	0.799

Data are expressed as mean ± SD.

^a,b,c^Values sharing the same superscript letter within the same row are not significantly different (*p* < 0.05).

### 3.5 SEM Results

**FIGURE 5:**
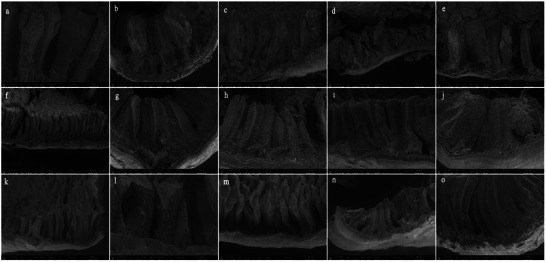
Representative SEM images showing villus morphology in the duodenum, jejunum, and ileum of control, sham, BB, DM, and DM+BB groups. Regular villus organization was observed in the control, sham, BB, and DM+BB groups, whereas diabetic rats exhibited irregular folding and surface structural alterations. All images were obtained at × 2000 magnification. Scale bar = 50 µm.

In the control, sham, BB, and DM+BB groups, the small intestinal villi exhibited a generally preserved mucosal surface architecture with regular alignment and intact structural organization across all intestinal segments. In contrast, the DM group showed marked alterations in villus morphology characterized by villus disorganization, epithelial disruption, and irregular folding in the duodenum, jejunum, and ileum (Figure 5).

Duodenum villi: (a) Control, (b) Sham, (c) BB, (d) DM, (e) DM+BB

Jejunum villi: (f) Control, (g) Sham, (h) BB, (ı) DM, (j) DM+BB

Ileum villi: (k) Control, (l) Sham, (m) BB, (n) DM, (o) DM+BB

No statistically significant difference in villus length was observed between the control and sham groups in any intestinal segment (*p* > 0.05). In the duodenum, the diabetes group exhibited significantly greater villus length than all other experimental groups (*p* < 0.05). In the jejunum, villus length was significantly increased in the diabetes group compared with the control, sham, and BB groups (*p* < 0.05), whereas the DM+BB group showed intermediate values without a consistent statistical separation from all groups. In the ileum, villus length was significantly higher in the diabetes group than in all other groups (*p* < 0.05). BB administration reduced the diabetes‐associated increase in villus length, with the most pronounced normalization observed in the ileum, while duodenal and jejunal villus lengths remained moderately elevated relative to control values (Table 6 and Figure 7).

**TABLE 6:** Comparison of duodenal, jejunal, and ileal villus length in control, sham, DM, BB, and DM+BB *Wistar albino* rats.

Groups	Duodenum villus length (µm)	Jejunum villus length (µm)	Ileum villus length (µm)	*p*
Control	277.60 ± 31.61^b^	221.98 ± 29.42^b^	132.02 ± 34.84^bc^	0.05
Sham	271.85 ± 32.95^bc^	217.19 ± 31.73^b^	122.73 ± 30.77^cd^	
DM	311.41 ± 38.19^a^	249.97 ± 52.19^a^	195.94 ± 38.80^a^	
BB	233.52 ± 27.62^d^	227.78 ± 30.66^b^	117.33 ± 24.72^d^	
DM+BB	260.24 ± 37.20^c^	231.85 ± 40.06^ab^	136.79 ± 41.70^b^	

^a,b,c,d^Different superscript letters in the same column indicate statistically significant differences between groups (*p* < 0.05).

Although diabetic rats exhibited increased villus length across all intestinal segments, histological examination revealed marked villus disorganization, epithelial disruption, and architectural distortion. These findings indicate that increased villus length was accompanied by marked architectural disruption and did not reflect preservation of normal mucosal organization. BB treatment attenuated these diabetes‐induced morphometric and architectural alterations and improved overall villus organization (Table 6 and Figures 5 and 7).

**FIGURE 6:**
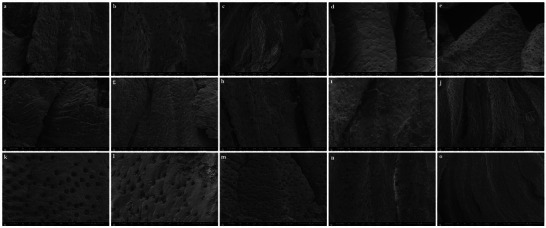
Representative histological images showing goblet cell morphology in the duodenum, jejunum, and ileum of control, sham, BB, DM, and DM+BB groups. Regular goblet cell distribution was observed in the control, sham, and BB groups, whereas the DM+BB group showed improved morphology compared with the DM group and a pattern closer to the control, sham, and BB groups. All images were obtained at × 2000 magnification. Scale bar = 50 µm.

In the control, sham, and BB groups, goblet cells in the duodenum, jejunum, and ileum exhibited regular morphology with uniform size and shape. In contrast, the DM group showed marked morphological alterations characterized by irregular shapes and increased size variability. In the DM+BB group, goblet cell morphology appeared improved compared with the DM group and showed a pattern closer to that observed in the control, sham, and BB groups (Figure 6).

**FIGURE 7:**
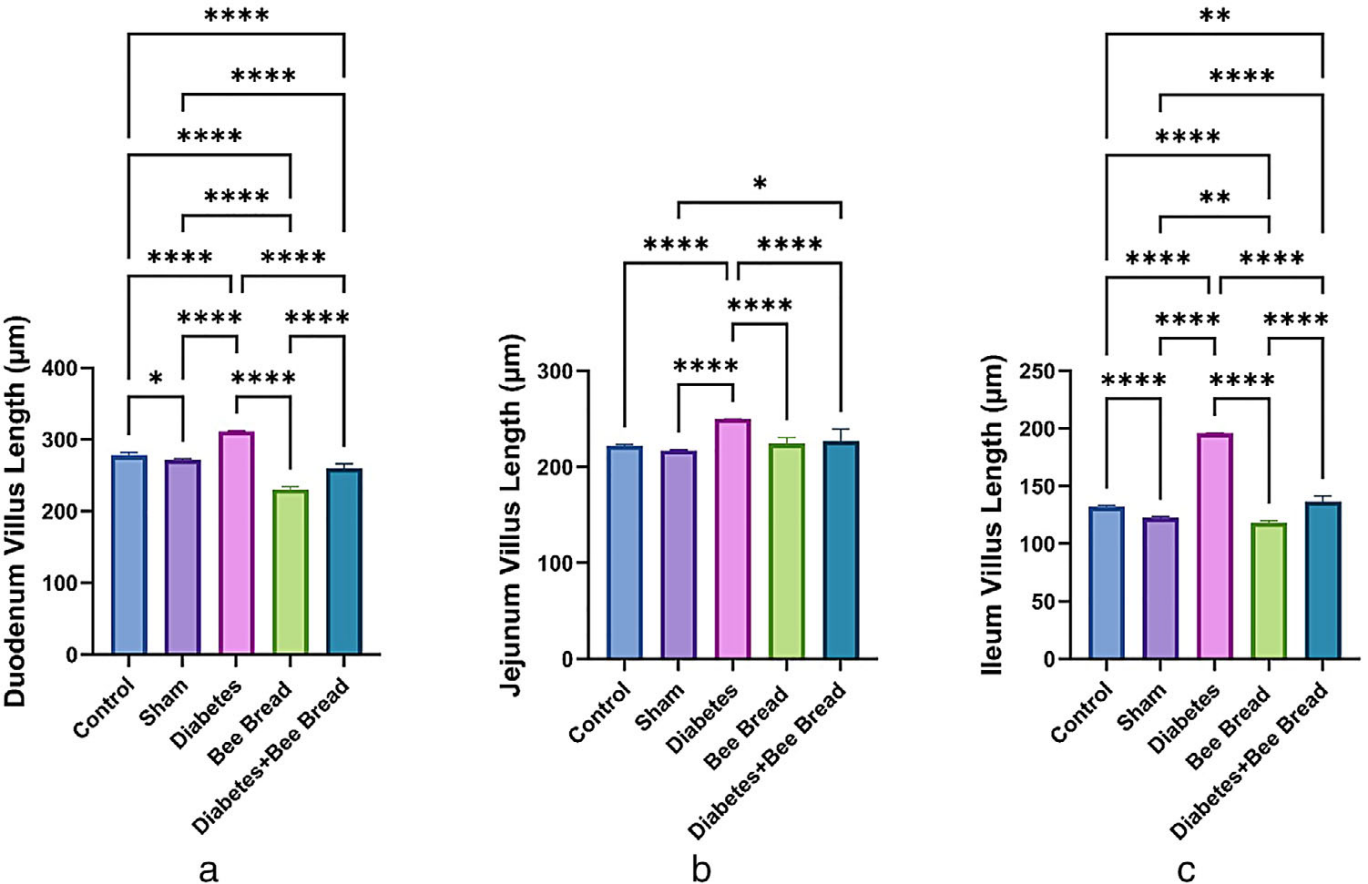
Comparison of villus length in the duodenum (A), jejunum (B), and ileum (C) of control, sham, DM, BB, and DM+BB groups. Villus length was significantly increased in diabetic rats across all small intestinal segments compared with the control and treatment groups, whereas BB and DM+BB treatments modulated diabetes‐induced alterations in villus morphometry. Data are presented as mean ± SD. Statistical analysis was performed using one‐way ANOVA followed by Tukey's post hoc multiple comparison test. ^*^ *p* < 0.05, ^**^ *p* < 0.01, ^***^ *p* < 0.001, ^****^ *p* < 0.0001.

Duodenum goblet cells: (a) Control, (b) Sham, (c) BB, (d) DM, (e) DM+BB

Jejunum goblet cells: (f) Control, (g) Sham, (h) BB, (ı) DM, (j) DM+BB

Ileum goblet cells: (k) Control, (l) Sham, (m) BB, (n) DM, (o) DM+BB.

## 4 Discussion

As hypothesized, BB administration protected the small intestinal mucosa and modulated Mn‐SOD‐associated antioxidant responses in STZ‐induced diabetic rats. BB is a nutrient‐dense and bioactive bee product whose composition depends on its botanical origin, geographic location, and storage conditions [21]. It contains proteins, essential amino acids, lipids, phenolic compounds, vitamins, minerals, carbohydrates, and flavonoids, many of which exhibit potent antioxidant and anti‐inflammatory properties [22, 23]. These bioactive components can modulate oxidative stress and support small intestinal barrier integrity. In addition, the anaerobic lactic fermentation process involved in BB formation enhances the bioavailability and digestibility of pollen‐derived nutrients, which may further contribute to its protective effects in intestinal tissues. The present findings are consistent with previous studies reporting that bee‐derived products can attenuate hyperglycemia‐induced oxidative stress and inflammation, thereby protecting intestinal structure and function in experimental diabetes models.

DM is strongly associated with systemic oxidative stress and diabetic enteropathy, a multifactorial gastrointestinal complication characterized by epithelial degeneration, villus disorganization, goblet cell dysfunction, impaired intestinal permeability, and disruption of intestinal homeostasis secondary to chronic hyperglycemia‐induced oxidative and inflammatory injury [24]. Hyperglycemia‐induced reactive oxygen metabolites (ROMs) disrupt the balance between pro‐oxidants and antioxidant defenses, leading to tissue damage. Excessive oxidative stress may further contribute to mitochondrial dysfunction, inflammatory signaling, and mucosal injury. Endogenous antioxidant enzymes, particularly SOD and CAT, constitute major components of the intestinal antioxidant defense system [5, 6].

In this study, Mn‐SOD immunoreactivity was observed predominantly in the muscularis, serosa, and crypt epithelial cells of diabetic rats, suggesting activation of a compensatory response to oxidative stress. Importantly, although overall Mn‐SOD immunoreactivity scores did not differ significantly among groups, diabetes altered the tissue distribution pattern of Mn‐SOD, particularly within crypt epithelial cells. This altered localization pattern represented one of the most important findings of the present study and may reflect increased oxidative burden within highly proliferative intestinal epithelial compartments. Therefore, the observed Mn‐SOD immunoreactivity should not be interpreted solely as enhanced antioxidant protection, but also as an indicator of adaptive cellular responses to ongoing oxidative stress. BB treatment appeared to modulate this altered distribution pattern, suggesting partial restoration of physiological redox organization within intestinal tissues. In contrast, CAT immunoreactivity remained weak across all groups, including diabetic animals. This discrepancy may reflect differential regulation of antioxidant enzymes; CAT may be less responsive during short‐term hyperglycemic intestinal injury, whereas alternative antioxidant pathways, such as glutathione peroxidase‐related systems, may contribute more prominently to hydrogen peroxide detoxification under these conditions [25, 26]. Weak CAT expression also highlights a limitation of relying solely on immunohistochemistry for enzyme assessment and underscores the importance of complementary quantitative biochemical assays in future studies.

BB supplementation partially mitigated DM induced intestinal damage. Histologically, the mucosal and muscular layers of the duodenum, jejunum, and ileum in the DM+BB group were preserved, villi exhibited regular morphology, and goblet cells maintained uniform shape and size. In contrast, the untreated DM group displayed villus disorganization, epithelial disruption, irregular alignment, and goblet cell heterogeneity. These findings suggest that BB exerts antioxidant effects in vivo, likely through phenolic and flavonoid‐mediated attenuation of oxidative stress, thereby maintaining tissue integrity and reducing mucosal injury [7, 27]. Previous studies have also suggested that bee‐derived products may exert anti‐inflammatory and anti‐apoptotic effects through modulation of inflammatory mediators and cellular stress responses. The incomplete normalization observed in the DM+BB group may reflect the relatively short duration of intervention, as well as the time‐dependent nature of metabolic and enzymatic recovery processes, which may require prolonged exposure to achieve full restoration of tissue homeostasis.

Goblet cell preservation is particularly important because goblet cells play a central role in mucin secretion and maintenance of intestinal barrier integrity. Alterations in goblet cell morphology may contribute to increased intestinal permeability and enhanced susceptibility to inflammatory injury. In the present study, diabetic animals exhibited marked goblet cell heterogeneity and morphological irregularities, whereas BB administration preserved goblet cell morphology and distribution. These findings suggest that the protective effects of BB extend beyond general tissue preservation and may contribute to maintenance of mucosal barrier organization.

Electron microscopy further supported the histological findings. Diabetic rats exhibited marked villus disorganization, irregular folding, and surface structural alterations throughout the intestinal tract, whereas BB administration largely preserved villus architecture and overall villus organization. These ultrastructural observations were consistent with the histopathological findings, which demonstrated improved mucosal integrity in BB‐treated diabetic animals. Collectively, these findings indicate that BB exerts protective effects not only at the histological level but also at the ultrastructural level. Preservation of villus architecture may contribute to maintenance of absorptive capacity and intestinal barrier function, both of which are commonly impaired in diabetes [28].

Although diabetes is generally associated with intestinal degeneration and barrier dysfunction [29], villus length was significantly increased in diabetic animals across all intestinal segments in the present study. Similar findings have been reported previously in STZ‐induced diabetic models and have been interpreted as adaptive intestinal remodeling characterized by villus elongation, increased epithelial turnover, and mucosal hyperplasia in response to chronic metabolic stress [30]. Therefore, increased villus length should not necessarily be interpreted as preservation of normal intestinal integrity, as villus elongation may coexist with epithelial degeneration, architectural distortion, and mucosal dysfunction [28]. Indeed, despite exhibiting greater villus length, diabetic animals in the present study showed marked villus disorganization and epithelial abnormalities. BB treatment partially attenuated these diabetes‐associated morphometric alterations and promoted restoration toward a more physiologically organized intestinal architecture.

The present study has several limitations. The relatively short 15‐day experiment period may have limited the ability to evaluate long‐term consequences. Enzyme assessment was based solely on immunohistochemistry without complementary biochemical quantification of SOD and CAT activity. Gut microbiota composition was not evaluated, precluding direct conclusions regarding potential microbiota‐mediated effects of BB. Furthermore, molecular pathways associated with inflammation, epithelial barrier regulation, and oxidative stress signaling were not investigated. Findings may also vary according to the botanical and geographical origin of BB. Future studies should extend the intervention duration, include quantitative enzymatic analyses, evaluate small intestinal barrier‐associated pathways, and incorporate microbiome investigations to elucidate the full spectrum of BB‐mediated protective mechanisms.

In summary, BB supplementation mitigated diabetes‐associated small intestinal injury and modulated antioxidant responses, predominantly associated with alterations in Mn‐SOD distribution and preservation of small intestinal morphology. The altered distribution pattern of Mn‐SOD in diabetic animals suggests localized oxidative stress responses within intestinal tissues, while the limited CAT responsiveness indicates that additional antioxidant mechanisms may contribute to hydrogen peroxide detoxification under hyperglycemic conditions. BB exerted protective effects at both histological and ultrastructural levels and partially attenuated diabetes‐associated intestinal remodeling. These findings support the potential of BB as a natural adjunctive strategy for alleviating diabetes‐associated small intestinal injury.

## 5 Conclusion

BB supplementation exerts protective effects on small intestinal tissue in diabetic rats by preserving mucosal and muscular integrity, maintaining regular villus architecture, and stabilizing goblet cell morphology. These findings indicate that BB provides a tissue‐protective effect against diabetes‐induced small intestinal injury, likely associated with modulation of endogenous antioxidant responses and maintenance of intestinal structural integrity. Taken together, the results support a local small intestinal protective role of BB rather than a systemic metabolic regulatory effect. Future studies, particularly translational and clinical investigations, are required to confirm these findings and to further clarify the molecular mechanisms underlying BB‐mediated small intestinal protection.

## Author Contributions

Ebru Karadag Sari participated in the study concept and design, methodology, investigation, formal analysis, data curation, and drafting of the manuscript. Buket Bakır contributed to the study concept and design, methodology, investigation, formal analysis, and revision of the manuscript. Sükran Yediel Aras and Sevda Elis Yıldız contributed to the investigation, data collection, and revision of the manuscript. Arzu Gezer contributed to the study validation and critical revision of the manuscript. All authors read and approved the final manuscript.

## Ethics Statement

The study was authorized by the Animal Experiments Ethics Committee of Tekirdağ Namık Kemal University (T2021‐662; 07.06.2021) and conducted in accordance with the Guide for the Care and Use of Laboratory Animals and the ARRIVE guidelines.

## Conflicts of Interest

The authors declare no conflict of interest.

## Declaration of Generative AI and AI‐Assisted Technologies in the Writing Process

AI‐based tools were used only for language editing and grammar correction.

## Data Availability

The data that support the findings of this study are available within the article.
